# Deep learning for malignant lymph node segmentation and detection: a review

**DOI:** 10.3389/fimmu.2025.1526518

**Published:** 2025-04-28

**Authors:** Wenxia Wu, Adrien Laville, Eric Deutsch, Roger Sun

**Affiliations:** Unité Mixte de Recherche (UMR) 1030, Gustave Roussy, Department of Radiation Oncology, Université Paris-Saclay, Villejuif, France

**Keywords:** deep learning, lymph node, segmentation, detection, delineation

## Abstract

Radiation therapy remains a cornerstone in the treatment of cancer, with the delineation of Organs at Risk (OARs), tumors, and malignant lymph nodes playing a critical role in the planning process. However, the manual segmentation of these anatomical structures is both time-consuming and costly, with inter-observer and intra-observer variability often leading to delineation errors. In recent years, deep learning-based automatic segmentation has gained increasing attention, leading to a proliferation of scholarly works on OAR and tumor segmentation algorithms utilizing deep learning techniques. Nevertheless, similar comprehensive reviews focusing solely on malignant lymph nodes are scarce. This paper provides an in-depth review of the advancements in deep learning for malignant lymph node segmentation and detection. After a brief overview of deep learning methodologies, the review examines specific models and their outcomes for malignant lymph node segmentation and detection across five clinical sites: head and neck, upper extremity, chest, abdomen, and pelvis. The discussion section extensively covers the current challenges and future trends in this field, analyzing how they might impact clinical applications. This review aims to bridge the gap in literature by providing a focused overview on deep learning applications in the context of malignant lymph node challenges, offering insights into their potential to enhance the precision and efficiency of cancer treatment planning.

## 1 Introduction

Radiation therapy stands as one of the most common modalities for cancer treatment, with more than 50% of cancer patients treated. Precise targeting and sparing of healthy tissues are paramount for its effectiveness. A critical step in planning radiation therapy is the delineation of anatomical structures in medical imaging, which includes the segmentation of the target volume (GTV, gross tumor volume) and organs at risk (OAR) (1). Proper delineation of these volumes is required to achieve the most of the tumor versus normal tissue differential effects of current high technologies image guided radiotherapy machines translating into improvements in tumor cure rates and minimization of side effects.

Traditionally, this task is achieved by radiation oncologists, relying heavily on manual delineation processes. However, these conventional methods are fraught with potential pitfalls, notably the subjective variability that can arise both within and between observers, potentially leading to inconsistencies in treatment planning.

**Figure 1:**
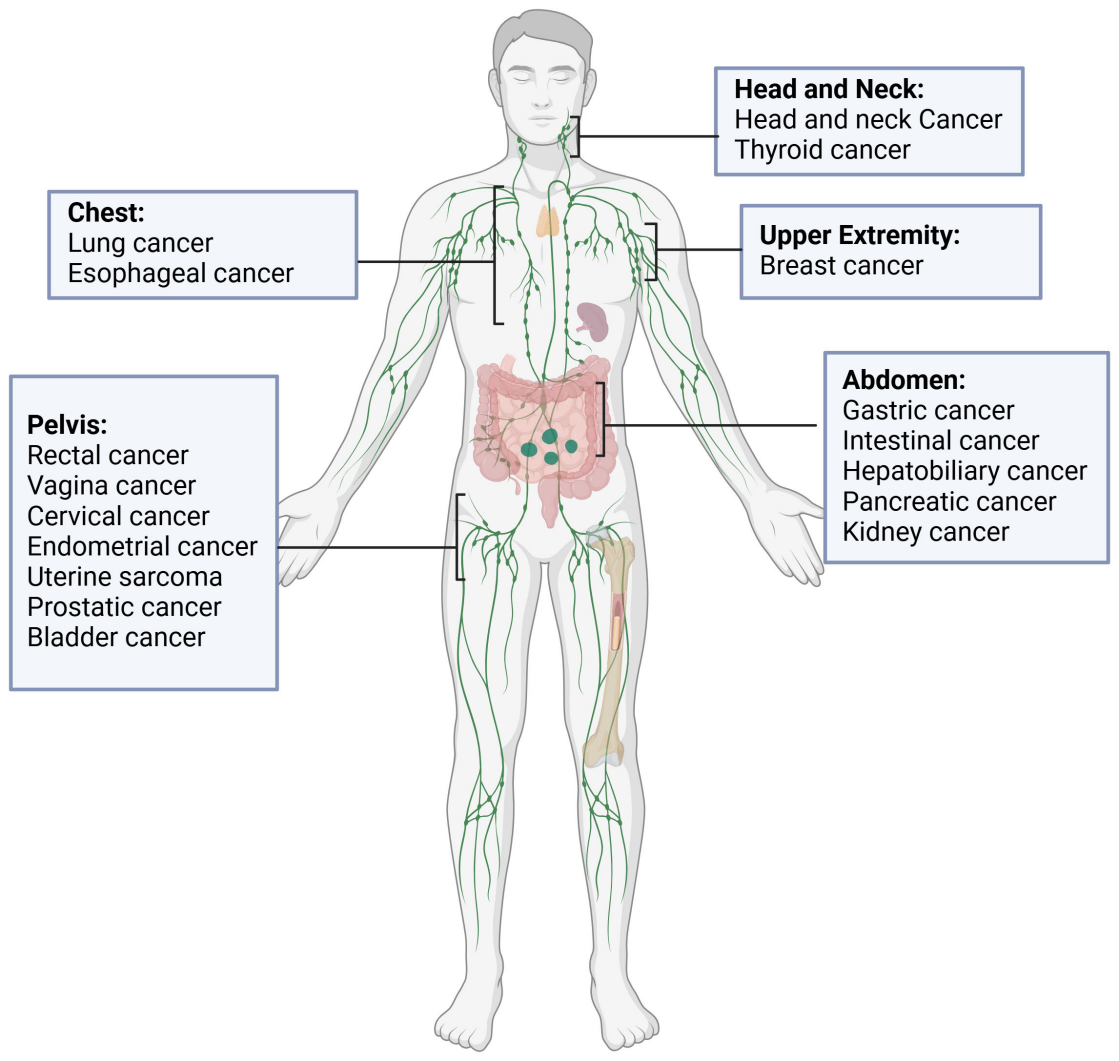
Representative tumors types involving lymph node metastasis in different anatomical regions.

In recent years, the advent of machine learning and deep learning technologies has heralded significant advancements in the field of automatic segmentation, offering promising solutions to overcome the limitations of manual segmentation. Notably, stateof-the-art (SOTA) algorithms have been developed for OAR segmentation (2), showcasing the potential of these technologies in enhancing the accuracy and efficiency of treatment planning. In fact, there are already commercially available, EMA and FDA-approved software solutions for the segmentation of normal tissues, based largely on machine learning (ML) and deep learning (DL) methodologies, like Annotate (3), Contour+ by MVision AI (4), Contour ProtégéAI by Mim Software (5), and Limbus Contour by Limbus AI (6). Moreover, comprehensive reviews have summarized the progress in tumor auto-segmentation in medical imaging. However, the automatic segmentation of metastatic lymph nodes (GTV N) remains an area relatively unexplored, despite its critical importance in cancer diagnosis and treatment. As shown in **Figure 1**, representative tumor types involving lymph node metastasis in different anatomical regions illustrate the heterogeneity of metastatic spread, further emphasizing the need for advanced segmentation techniques.

Lymph nodes and tumor-infiltrated lymph nodes present distinct challenges in medical imaging. Lymph nodes can be classified as either tumor-negative (benign) or tumor-positive (malignant), and this distinction is crucial in clinical practice. Benign lymph nodes function as part of the immune system and are classified as organs-at-risk (OARs), requiring protection during radiotherapy to avoid unnecessary damage. In contrast, malignant or tumor-infiltrated lymph nodes are potential therapeutic targets, as they may harbor metastases (7) and thus need to be identified and treated, often through surgical removal or radiation.

Additionally, conventional imaging modalities such as CT, MR, and PET/CT frequently fail to reliably detect or distinguish tumor-positive lymph nodes when their diameter is less than 10-12mm. This presents a further complication in the segmentation task, as it is difficult to discern small malignant nodes from benign ones based solely on imaging features. Consequently, more advanced methods are needed to ensure accurate differentiation and treatment planning, ensuring that malignant nodes are targeted, while benign lymph nodes are preserved as OARs.

The analysis of lymph nodes, particularly in identifying and segmenting malignant ones, is crucial for cancer staging, prognosis assessment, and treatment planning. As key components of the immune system, lymph nodes serve as sentinels, signaling the presence of cancerous cells (8).

In radiotherapy, automatic segmentation can shorten the planning workflow and improve reproducibility. (9, 10). Beyond radiotherapy, lymph nodes are also central to the success of immunotherapies, including immune checkpoint inhibitors (ICIs), which have significantly refined cancer management. However, despite their effectiveness (11, 12), only a fraction of patients achieve a sustained response (13). This variability is influenced by the complex interactions between the immune system and tumors, where lymph nodes play a vital role in T cell priming and activation. Radiomics analysis of lymph nodes, with accurate autosegmentation, could help to better understand factors implied in ICI response (14–16). For example, identifying lymph nodes with T-cells exposed to responsive antigens could be crucial for targeting lesions in stereotactic body radiotherapy (14, 17).

Lymph node dissection is invasive and induce morbidity. It’s controversial if lymph node dissection is critical for tumor response to ICI (18, 19). Accurate segmentation of lymph nodes could improve patient selection for nodal classification in several cancers (20). Tumor draining lymph nodes are involved in immune priming responsible of tumor control (12, 21, 22) could help to explore these regions to improve radiation and immunotherapy combination (11). Enhanced targeting of pathological lymph nodes could allow sparing the unaffected lymphatic system, potentially reducing lymphopenia and significantly impacting patient outcomes (23).

**Figure 2:**
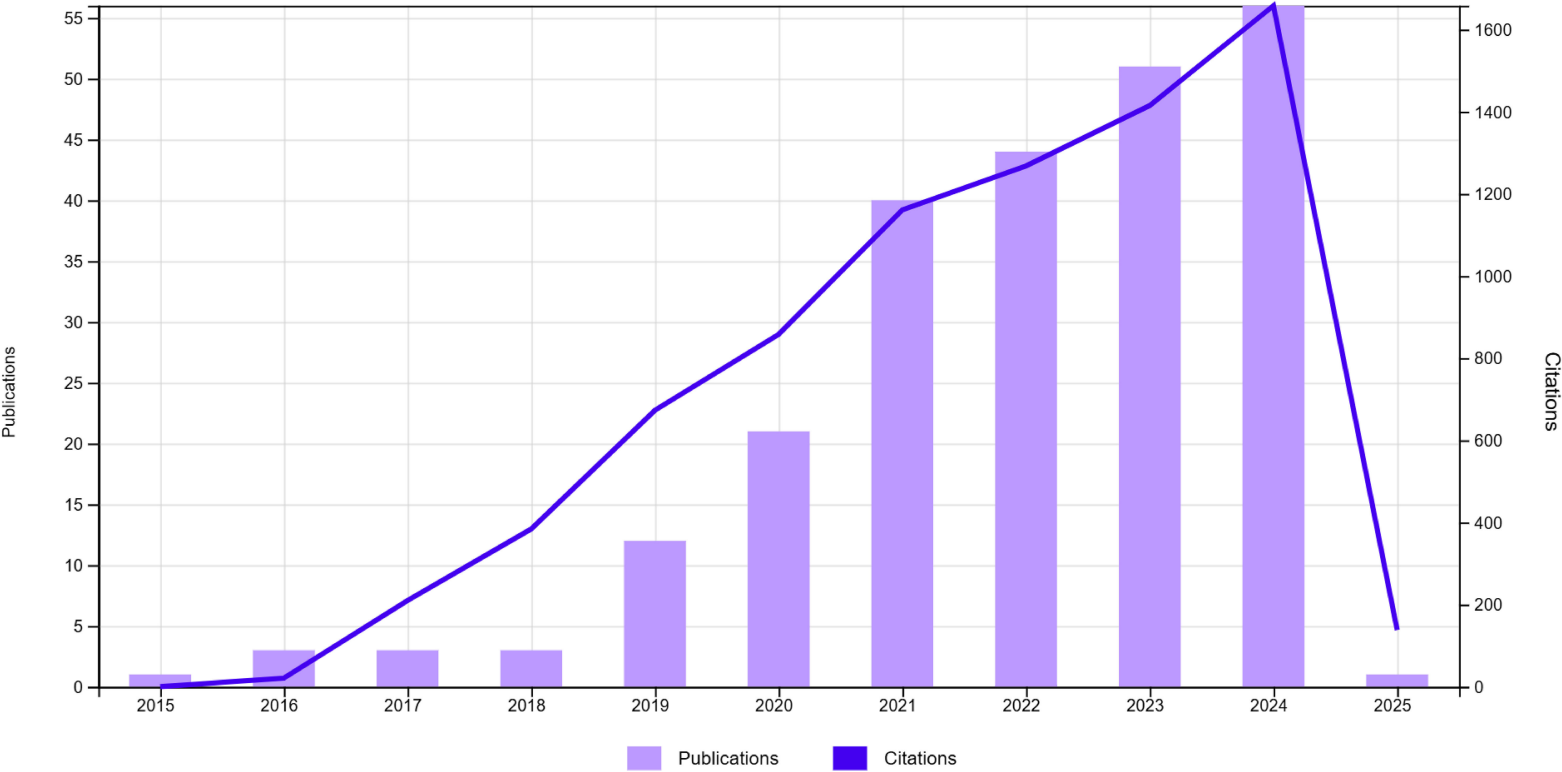
Overview of numbers of papers published and cited from 2015 to 2024 regarding deep learning lymph node segmentation.

In recent years, several works based on deep learning have been proposed to address the inherent difficulties associated with lymph node analysis in medical imaging 1, as shown in **Figure 2**. However, these methods still face a multitude of challenges. Beyond the issue of limited data availability, the segmentation and detection of malignant lymph nodes present unique problems, which include:(a)Uncertainty of the prediction of involved lymph node, especially for small adenopathies;(b) Variations in the shape, location, and size of lymph nodes add complexity to the segmentation process. Normal lymph nodes typically exhibit a regular ovoid shape, which can be clearly distinguished in imaging scans. In contrast, malignant lymph nodes often deviate from this regular form, displaying irregular, enlarged, or lobulated contours, further complicating their accurate detection and differentiation;(c) Lymph nodes can easily be mistaken for blood vessels or other anatomical structures due to their similar appearance in imaging scans;(d) Issues such as aliasing, sampling, reconstruction, and noise during the image acquisition process compromise image quality;(e) The boundaries of lymph nodes may be indistinct, especially when they are adjacent to diseased tissues or have been invaded by tumor cells. These factors collectively underscore the complexity of developing robust algorithms for lymph node analysis in the field of medical imaging.

Traditional segmentation methods, such as thresholding, region growing, and edge detection (24), have historically been used for lymph node segmentation. In medical imaging, atlas-based segmentation (25) has also been widely applied, where a pre-defined anatomical model is used to guide the segmentation process. While atlas-based methods provide structural guidance, they still struggle with the challenges of complex medical images, such as irregular shapes, variable lymph node sizes, and unclear boundaries. These traditional approaches rely heavily on basic features, making them inadequate for precise lymph node segmentation in clinical applications.

Machine learning-based approaches, such as random forests and support vector machines (SVM) (26), have shown improvement over these methods by leveraging more advanced feature sets. However, they still rely on handcrafted features and often face difficulties in generalizing across different datasets and imaging conditions.

To address these limitations, deep learning, particularly U-Net (27), has emerged as a powerful tool in medical image segmentation, offering the capability to automatically learn complex, high-level features directly from data. U-Net’s encoderdecoder architecture captures both local and global features, enhancing segmentation accuracy. Additionally, deep learning’s automated feature extraction and adaptability make it highly effective for varied data and complex anatomical cases. Despite interpretability challenges, deep learning remains dominant due to its superior accuracy, automated feature learning, and adaptability across diverse imaging conditions. Recent tools like Class Activation Mapping (CAM) have also improved interpretability, maintaining its clinical relevance as the primary method for medical image segmentation.

The primary aim of this review is to assess the current state of deep learning-based techniques for the detection and segmentation of malignant lymph nodes in medical imaging. Specifically, it seeks to evaluate the performance of different deep learning models, identify challenges and limitations in current research, and suggest future directions for study. By bridging the gap between traditional methods and the latest technological advancements, this review aspires to enhance the outcomes of cancer diagnosis and treatment, ultimately contributing to improved patient care and survival rates.

## 2 Deep learning medical image segmentation

### 2.1 Basis of deep learning

**Figure 3:**
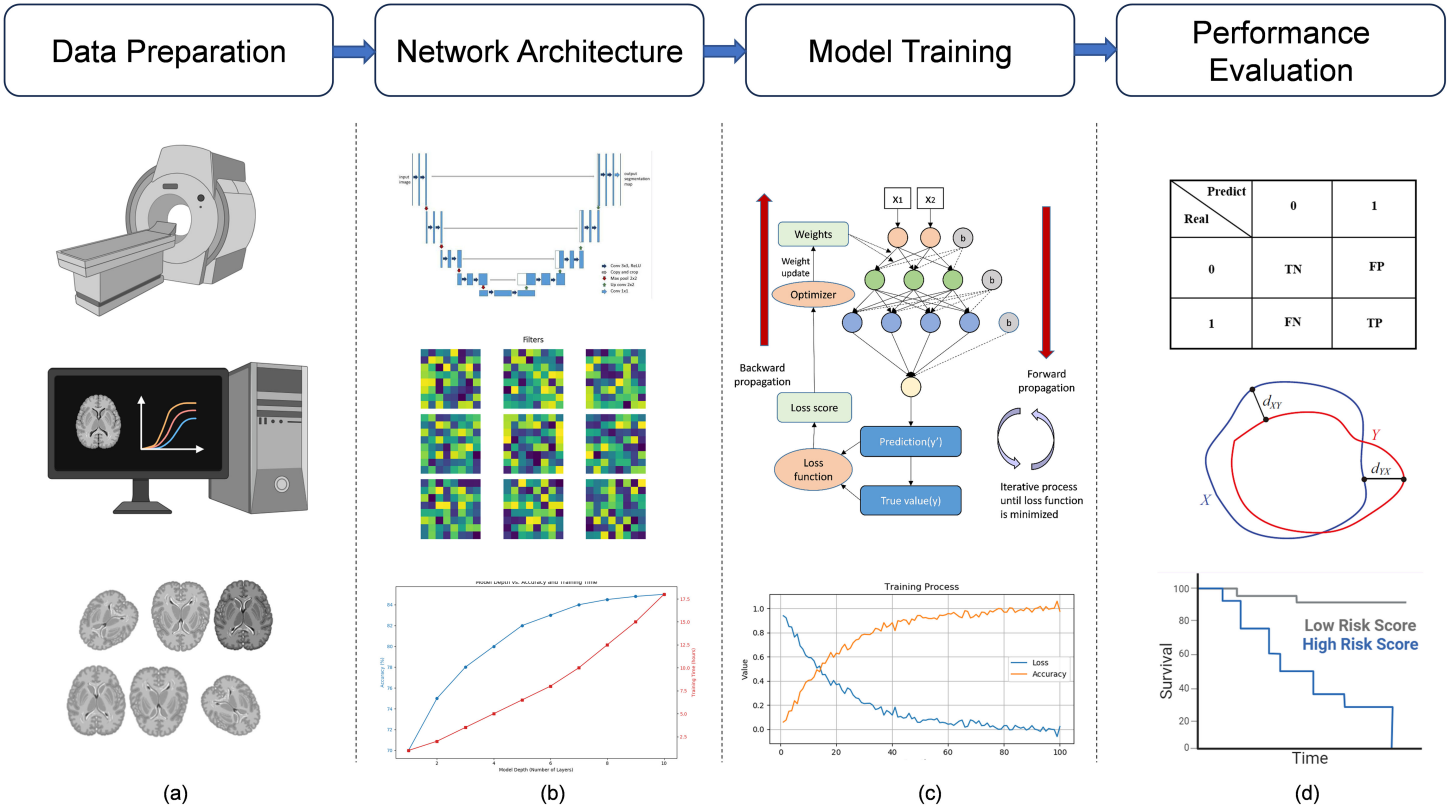
Deep learning medical image analysis pipeline. Deep learning medical image analysis pipeline. **(a)** MRI images are acquired, filtered, analyzed, and preprocessed (e.g., normalization, augmentation) to ensure data quality and consistency before model input. **(b)** Based on the task and data characteristics, an appropriate deep learning model is constructed, such as the U-Net, for medical image analysis. **(c)** The model is trained with labeled data, optimized iteratively through forward and backward propagation (e.g., gradient descent), and validated to assess performance. **(d)** The trained model is tested on unseen data (test set), and results are evaluated using metrics such as confusion matrices, ROC curves, and survival analysis to assess predictive power.

Deep learning, a specialized branch of machine learning, is distinguished by its capacity to extract features from raw data autonomously. This capacity is notably augmented by the network’s depth, facilitating the formulation of more intricate feature hierarchies. Deep learning has garnered significant scholarly interest in medical image segmentation, attributed to its adeptness in accurately demarcating precise anatomical configurations from medical imagery. The conventional workflow in this field is comprised of several critical stages: data preparation, network architecture development, model training, and performance evaluation, as shown in **Figure 3**. Each of these stages is foundational to the construction of effective segmentation solutions.

#### 2.1.1 Data preparation

This initial phase involves the collection, preprocessing, and augmentation of medical images to create a comprehensive dataset. Image preprocessing techniques such as normalization, noise reduction, and contrast enhancement are employed to improve the quality of the images (28). These techniques ensure that the images are standardized, clearer, and more suitable for analysis. Following preprocessing, data augmentation strategies, including rotation, scaling, and flipping, are utilized to increase the diversity of the dataset. This augmentation enhances the model’s ability to generalize by exposing it to a wider variety of image transformations, which can help in improving its robustness and accuracy (29).

#### 2.1.2 Network architecture

At this juncture, the focus is on designing a neural network architecture that is adept at capturing the complexities of medical images. This process involves selecting the appropriate type of neural networks, such as Convolutional Neural Networks (CNNs) or Transformer-based models, based on the specific requirements of the medical imaging task, as illustrated in **Table 1**. The configuration of the network’s layers and connections is crucial to optimizing feature extraction and segmentation accuracy. Key considerations include the number and type of layers (e.g., convolutional layers, pooling layers, fully connected layers), activation functions, and the network’s depth. The architecture must balance computational efficiency with the need for deep, intricate learning structures, ensuring it can effectively process high-dimensional data while maintaining practical training and inference times.

**Table 1:** Comparison between CNNs and transformer-based models.

Aspect	CNNs based Models	Transformer-based Models
Architecture	Uses convolutional layers to capture spatial hierarchies and patterns	Utilizes self-attention mechanisms to capture long- range dependencies.
Core Mechanism	Convolutions over local regions (filtering and pooling)	Self-attention: each token attends to every other token in a sequence.
Main Application Areas	Image classification, object detection, image segmentation.	Natural language processing, translation, text generation, image understanding (e.g., Vision Transformers)
Computational Complexity	*O*(*n*) per convolutional layer for image size *n* × *n*. Complexity increases with depth and larger filters.	*O*(*n* ^2^) due to self-attention, where *n* is the input length. Scales quadratically with sequence length.
Receptive Field	Limited to local regions, expands with depth but requires multiple layers for global context.	Global receptive field from the first layer due to self-attention. Each token attends to all others directly.
Representative Models Efficiency	AlexNet, VGG, ResNet, EfficientNet Computationally efficient for smaller images and tasks due to local filters.	BERT, GPT, Vision Transformer (ViT), BART. Requires more compute and memory due to the self-attention mechanism.
Receptive Field	Limited to local regions, expands with depth but requires multiple layers for global context.	Global receptive field from the first layer due to self- attention. Each token attends to all others directly.
Inductive Bias	Strong spatial locality bias (fixed receptive fields)	No inherent spatial or sequential bias (fully data- driven)
Multimodal Task Adaptability Scalability	Less naturally suited for multimodal tasks, but can be adapted. Scales efficiently with respect to spatial data but may struggle with long-range dependencies	Well-suited for multimodal tasks, such as combining vision and language Scales effectively for large datasets and long sequences due to parallelizable self-attention
Training Difficulty	Typically easier to train, especially on smaller datasets. Pretraining on large datasets not always necessary.	Requires large-scale datasets and pretraining to achieve good performance. Training can be more challenging due to sensitivity to hyperparameters and large memory consumption.
Interpretability	Generally more interpretable due to local operations of convolutional filters. Feature maps can be visualized to understand spatial patterns.	Harder to interpret due to global self-attention mechanism. Attention maps provide some insight, but the model’s decision process is often opaque.

#### 2.1.3 Model training

The training process involves optimizing the network’s weights through the iterative processing of the prepared dataset. This phase employs backpropagation and gradient descent algorithms to minimize the loss function, which measures the discrepancy between the predicted segmentation and the ground truth. The choice of optimizer (such as Adam or SGD), learning rate, and regularization techniques (such as dropout or weight decay) plays a pivotal role in ensuring the model converges to a solution that generalizes well to unseen data. Proper tuning of these hyperparameters is crucial for balancing the trade-off between underfitting and overfitting, ultimately enhancing the model’s performance on new, unseen medical images.

#### 2.1.4 Performance evaluation

The performance evaluation phase assesses the model’s effectiveness using task-specific metrics. For classification tasks, metrics such as accuracy, precision, recall, and the F1 score are used, alongside a confusion matrix to detail true and false classifications. Segmentation tasks often use the Dice coefficient, Intersection over Union (IoU), and pixel accuracy to measure overlap and correct pixel classification. Detection tasks rely on mean Average Precision (mAP), precision, and recall to evaluate performance. **Table 2** outlines common segmentation and detection metrics. These metrics ensure the model’s robustness and accuracy, guiding further optimization to enhance its generalization to unseen data.

**Table 2:** Segmentation and detection performance metrics.

(a) Segmentation Metrics		
Category	Metric Name	Formula
Overlap-based	Accuracy	$Acc=\frac{\left|TP+TN\right|}{\left|TP+FP+FN+TN\right|}$
	Precision (PPV)	$PPV=\frac{TP}{TP+FP}$
	Recall (Sensitivity)	$Sens=\frac{TP}{TP+FN}$
	F1-score	$F1\;=\;2\times \frac{Precision\times Recall}{Precision+Recall}$
	Intersection over Union (IoU)	$IoU=\frac{\left|G\cap S\right|}{\left|G\cup S\right|}$
	Dice Similarity Coefficient (DSC)	$DSC=\frac{2\left|G\cap S\right|}{\left|G\right|+\left|S\right|}$
	Absolute Volume Difference (AVD)	$AVD=\frac{\left|V_{p}-V_{g}\right|}{V_{g}}\times 100$
	Hausdorff Distance (HD)	$HD=\max\left\{\sup_{x\in G}\inf_{y\in S}d(x,y),\text{su}{\text{p}}_{y\in S}\inf_{x\in G}d(x,y)\right\}$
Distance-based	Average Hausdorff Distance (AHD)	$AHD=\frac{1}{2}\left(\frac{1}{\left|P\right|}\sum_{p\in P}\inf_{g\in G}d(p,g)+\frac{1}{\left|G\right|}\sum_{g\in G}\inf_{p\in P}d(p,g)\right)$
	Boundary-based F1-score	$F1_{bound}=2\times \frac{Precision_{bound}\times Recall_{bound}}{Precision_{bound}+Recall_{bound}}$

(b) Detection Metrics		
Metric Name	Formula	Explanation
Precision (PPV)	$PPV=\frac{TP}{TP+FP}$	Measures prediction reliability.
Recall (Sensitivity)	$Recall=\frac{TP}{TP+FN}$	Measures model’s ability to capture true positives.
F1-score	$F1\;=\;2\times \frac{Precision\times Recall}{Precision+Recall}$	Harmonic mean of Precision and Recall.
Intersection over Union (IoU)	$IoU=\frac{\left|G\cap P\right|}{\left|G\cup P\right|}$	Measures overlap between predicted and true boxes.
Average Precision (AP)	$AP=\int_{0}^{1}p\left(r\right)\;dr$	Precision-Recall curve’s area under the curve (AUC).
mean Average Precision (mAP)	$mAP=\frac{1}{N}\sum_{i=1}^{N}AP_{i}$	Average of AP across all classes.
GIoU (Generalized IoU)	$GIoU=IoU-\frac{\left|C-\left(G\cup P\right)\right|}{\left|C\right|}$	Considers both IoU and spatial relationship of boxes.

### 2.2 Deep learning models for medical image segmentation

The evolution of deep learning models has revolutionized medical image segmentation, offering unprecedented accuracy and efficiency. These models can be categorized into three primary classes: CNN-based models, Transformer-based models, and other innovative approaches.

#### 2.2.1 CNN-based models

**Figure 4:**
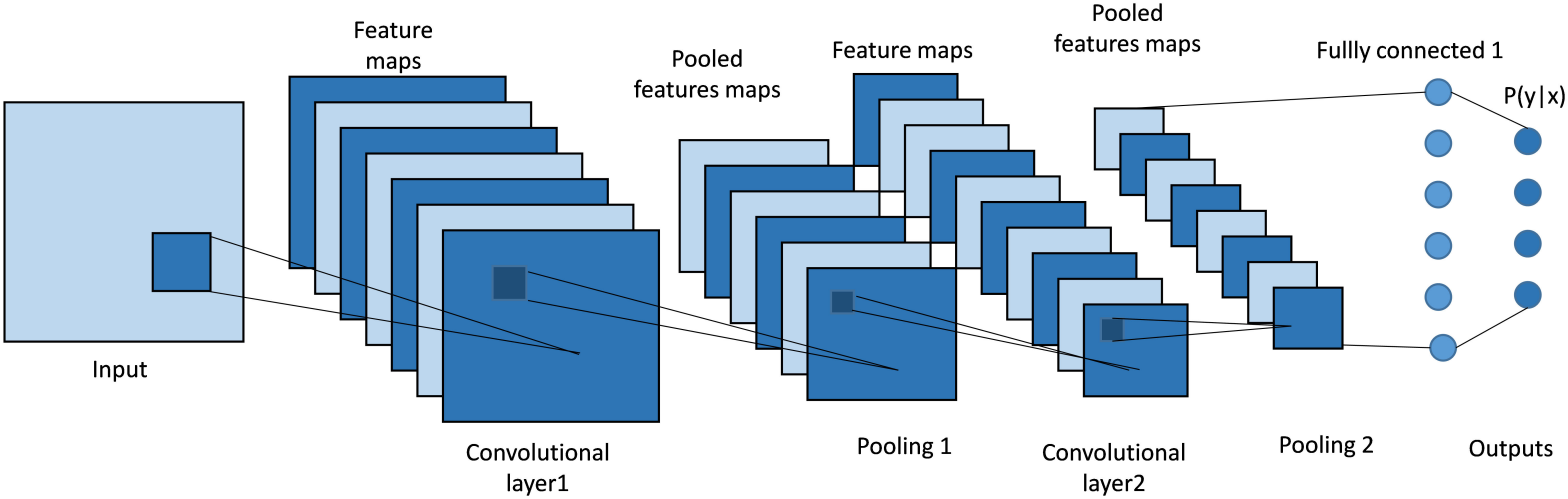
Architecture of a classical CNN. It uses convolutional layers with learnable filters to automatically extract spatial features and hierarchical patterns from input data.

Convolutional Neural Networks (CNNs), as shown in **Figure 4** are a type of deep learning model commonly used in image recognition tasks. At their core, CNNs use a mathematical operation called convolution. Convolution involves sliding a small matrix, known as a filter or kernel, over an image. At each position, the filter performs element-wise multiplication with the overlapping pixels, then sums these products to produce a single value. This process creates a feature map, which highlights important features like edges and textures in the image. By stacking multiple convolutional layers, CNNs can learn to detect complex patterns, making them highly effective for tasks such as identifying objects in images.

**Figure 5:**
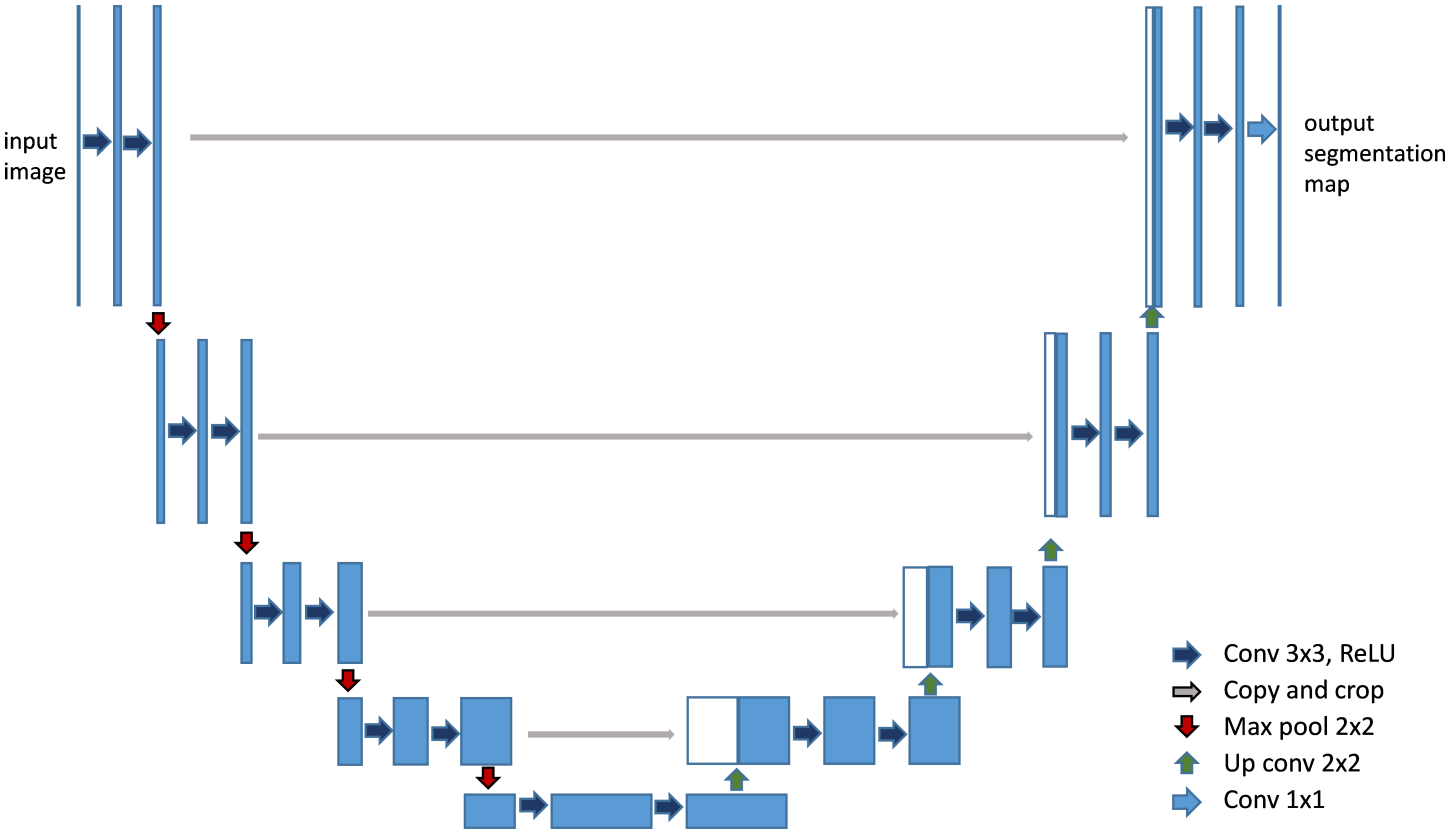
Architecture of U-Net (27). It features a symmetric U-shaped structure with an encoder to capture context and a decoder to enable precise localization.

CNN-based models, such as FCN (Fully Convolutional Networks) (30) and U-Net (27), have been instrumental in establishing the groundwork for medical image segmentation. These models leverage an architecture that integrates convolutional layers, pooling, and upsampling techniques. Such a design is adept at capturing spatial hierarchies and feature maps, making them highly effective for segmentation tasks. FCN pioneered the use of fully convolutional networks for pixel- wise segmentation, demonstrating significant improvements over traditional methods that relied on fully connected layers for classification tasks. With its unique encoder-decoder structure and skip connections, as shown in **Figure 5**, U-Net (27) effectively captures both context and localization information, making it especially suitable for medical image segmentation where precision is critical. Its variants, like 3D U-Net (31) and U-Net++ (32), further extend its applicability to volumetric data and enhance segmentation accuracy through architectural innovations (33).

#### 2.2.2 Transformer-based models

**Figure 6:**
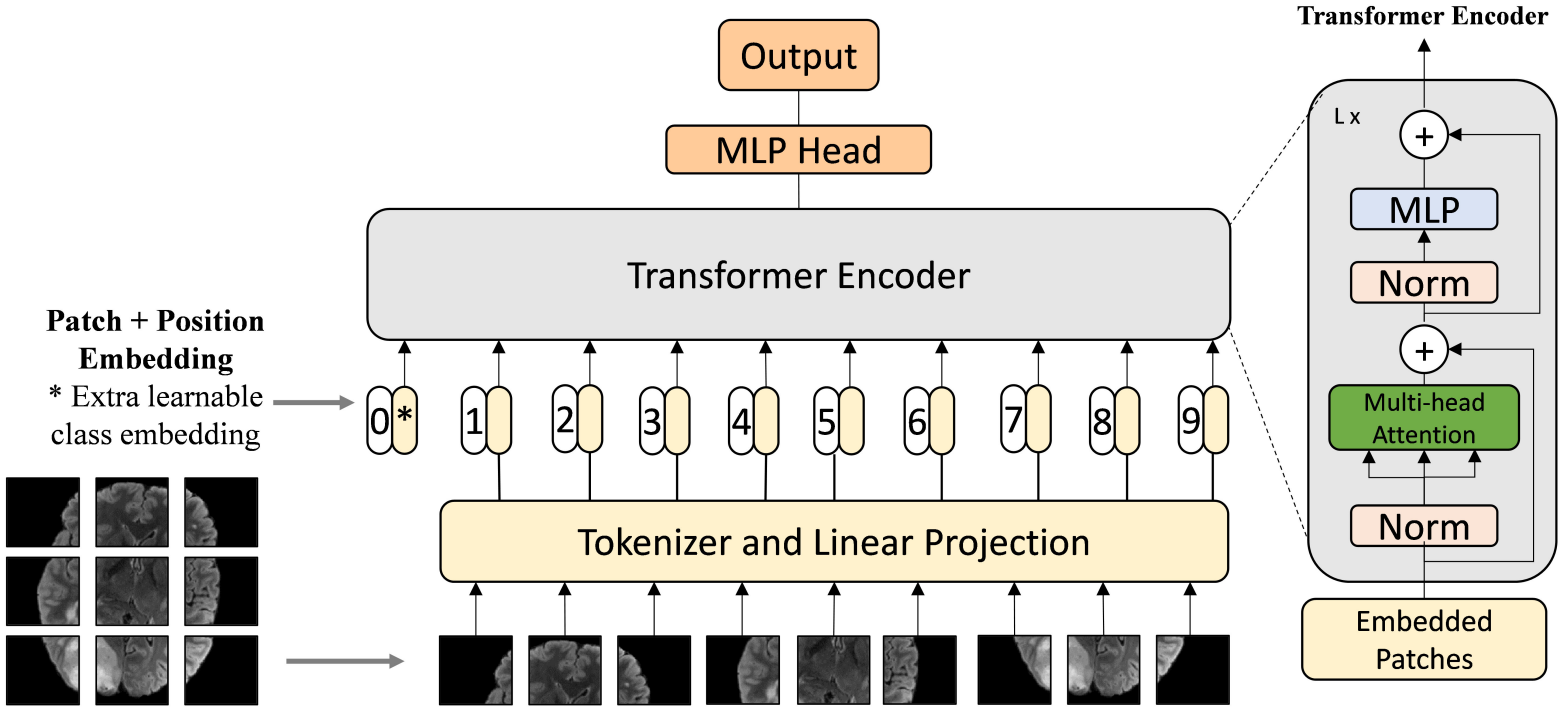
Architecture of Vision Transformer (34). The Vision Transformer divides an image into patches and applies self-attention mechanisms to model relationships between them using Transformer architecture.

Vision Transformers (ViTs) (34) represent a significant advancement in the field of computer vision, taking inspiration from the success of Transformers in natural language processing. Unlike traditional Convolutional Neural Networks (CNNs), which rely on convolutional layers to extract local features from images, Vision Transformers leverage a self-attention mechanism to capture global relationships between different parts of an image simultaneously, as shown in **Figure 6**. Instead of processing images through localized filters, ViTs split the image into fixed-size patches and use self-attention to model the dependencies between these patches across the entire image.

The self-attention mechanism in Vision Transformers operates through the following key steps:

1. Calculating Attention Weights: For each image patch, the model generates three vectors: Query (Q), Key (K), and Value (V). The Query vector represents the patch in focus, while the Key and Value vectors represent all other patches in the image. The similarity between the Query and each Key is calculated using a dot product, producing a set of raw attention weights *W*.
2. Normalization: The raw attention weights *W* are passed through a softmax function, ensuring that the weights are normalized and sum to 1. This step determines how much attention the model should pay to each patch in relation to the current patch.
3. Weighted Sum of Values: The normalized attention weights are multiplied by the corresponding Value vectors of the patches. The resulting products are summed to produce the final attention output for the patch, allowing the model to capture contextual information from the entire image.

**Figure 7:**
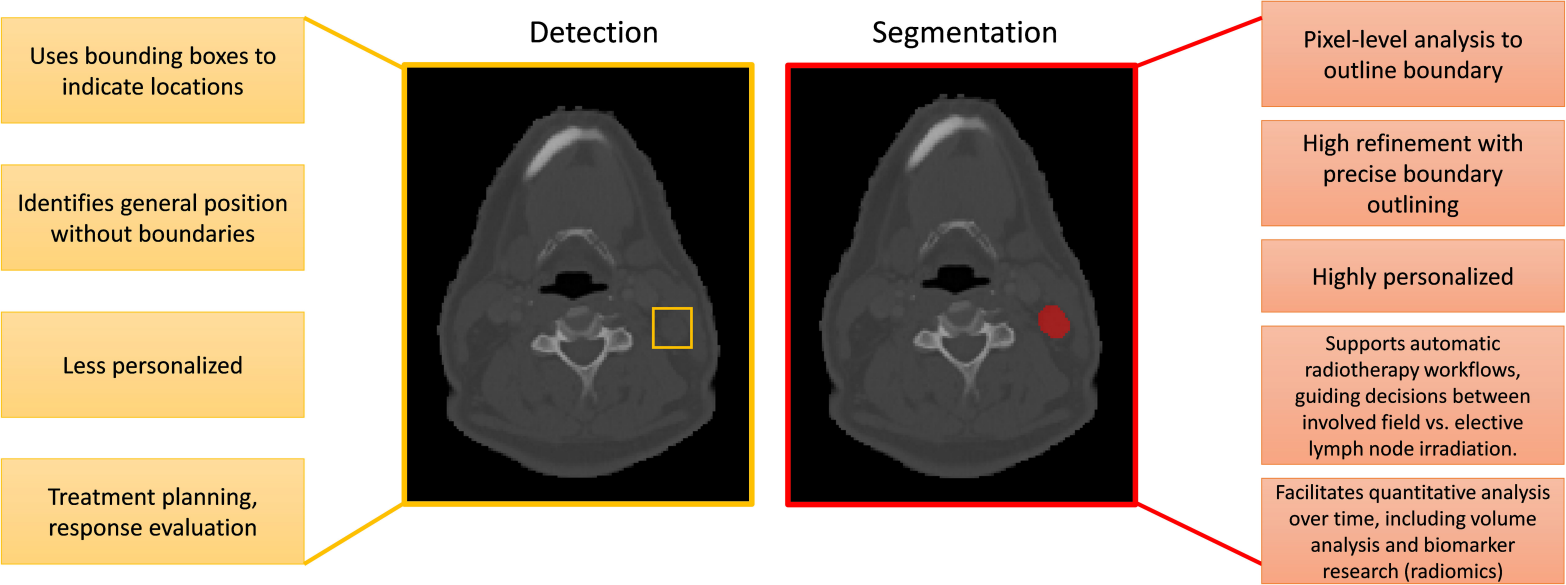
Summary of the differences between lymph node segmentation and detection.

After applying self-attention to the feature map *X*, a residual connection is typically added to retain the original information while incorporating the global context: *Y* = *X* +self_attention(*X*), as is shown in **Figure 7**. This integration allows Vision Transformers to capture complex relationships across the entire image, providing a powerful alternative to traditional CNNs.

The advent of Transformer-based models, such as TransUnet (35) and Swin-Unet (36), represents a pivotal leap forward in the domain of medical image segmentation. By harnessing self-attention mechanisms, these models adeptly model long-range dependencies, addressing the CNNs’ limitations in capturing global context—a crucial factor in comprehending the complexities of medical images. The self-attention mechanism works by weighing the importance of different input tokens relative to each other, allowing the model to focus on the most relevant parts of the input when making predictions.

Transformer-based models excel in modeling spatial relationships and feature interactions throughout the image, facilitating a deeper and more nuanced understanding of medical images. This enhanced capability has been instrumental in their successful deployment across a variety of segmentation tasks, where they have demonstrated significant improvements in delineating fine details and intricate structures. TransUnet (35) exemplifies this progress by melding the robust feature extraction capabilities inherent in CNNs with the Transformer’s adeptness at modeling long-range dependencies. This fusion establishes new benchmarks in segmentation performance. On the other hand, SwinUnet (36) introduces a hierarchical Transformer architecture that mirrors the inherent structure of medical images. This alignment significantly boosts both efficiency and accuracy in segmentation tasks, showcasing the transformative potential of these models in medical image analysis.

#### 2.2.3 Other models

The pursuit of more advanced medical image segmentation techniques has driven the exploration of novel architectures beyond traditional CNNs and Transformers, each offering unique mechanisms and distinct advantages.

Generative Adversarial Networks (GANs) (37) consist of two main components: the generator and the discriminator. The generator creates synthetic images, while the discriminator evaluates them against real images to improve the generator’s performance over time (38). There are several methods to enhance segmentation performance using GANs. Firstly, GANs address issues of multi-center datasets, imbalance, incompleteness, and poor quality in medical imaging by generating realistic synthetic images. These synthetic samples are used to augment existing datasets (39), effectively enhancing the training of segmentation models and improving their performance. Commonly used approaches include CycleGAN (40) for unpaired data and Pix2Pix (41) for paired data. Secondly, the concept of adversarial training inspired by GANs has been utilized to enhance segmentation methods. In this approach, the segmentation network is trained to produce accurate segmentation maps, while the discriminator distinguishes between the predicted maps and the actual ground truth maps. The adversarial loss helps the segmentation network refine its predictions to be more accurate and realistic. Several studies have demonstrated that adversarial training and adversarial loss can significantly improve the performance of segmentation models.

State Space Models (SSMs) (42), particularly the Mamba model (43) with selection mechanisms and hardware-aware architecture, have recently garnered significant interest in sequential modeling and visual representation learning. These models challenge the dominance of transformers by providing infinite context lengths and maintaining substantial efficiency with linear complexity relative to the input sequence. Numerous studies have explored applications based on this model. For instance, Wang et al. introduced Mamba-UNet (44), a novel architecture that combines the strengths of the U-Net (27) in medical image segmentation with Mamba’s capabilities. Mamba-UNet (44) adopts a VMamba-based encoder-decoder structure, incorporating skip connections to retain spatial information across different network scales. Additionally, hybrid structures like U-Mamba have been developed, combining CNNs and SSMs to form a robust framework. This hybrid approach leverages CNNs’ proficiency in extracting local features and SSMs’ capacity to capture extensive relationships within images. Structured with an encoder-decoder setup, this architecture enhances its effectiveness in managing long-range data and adapts well to diverse segmentation tasks.

Vision Foundation Models (VFMs) are pre-trained on large-scale datasets using selfsupervised or semi-supervised learning, enabling their adaptation to various downstream tasks. Examples include CLIPSeg (45), SegGPT (46), and SAM (47). Recently, SAM has been widely explored in medical image segmentation. MedSAM (48) fine-tunes SAM on extensive medical segmentation datasets, extending its applicability to general medical image segmentation. It outperforms SAM on 21 3D and 9 2D segmentation tasks. AutoSAM (49) introduces a fully automated prompt generation solution for SAM, where an auxiliary encoder network generates alternative prompts based on input images. With fewer trainable parameters, AutoSAM achieves comparable segmentation performance. In ophthalmic multi-target segmentation, a fine-tuned SAM with a learnable prompt layer accurately segments structures such as vessels, lesions, or retinal layers across different imaging modalities. Medical SAM Adapter (50) is specifically designed for medical image segmentation, accommodating the high-dimensional nature (3D) of medical data and incorporating unique visual prompts like points and boxes. These advancements demonstrate the potential of VFMs in enhancing medical image analysis.

### 2.3 Loss functions

Cross-Entropy Loss (CE) stands as a cornerstone for evaluating the discrepancy between the predicted probabilities and the ground truth labels across multiple classes. It is formally expressed as:


(1) $$L_{CE}=-\frac{1}{N}\sum_{i=1}^{N}\sum_{c=0}^{C}g_{ci}\log(p_{ci})$$

where *N* denotes the total number of pixels, *C* the class count, *g_ci_* the ground truth, and *p_ci_* the predicted probability for class *c* at pixel *i*. This loss function is particularly adept at handling problems with multiple classes but requires adjustments, such as Weighted Cross-Entropy or Focal Loss, to effectively manage class imbalance issues prevalent in medical datasets.

Dice Loss, derived from the Sørensen-Dice coefficient, emphasizes the overlap between the predicted segmentation and the ground truth, making it especially suitable for datasets where positive samples are scarce. The loss is computed as:


(2) $$L_{D}=1-\frac{2\sum_{i=1}^{N}g_{i}p_{i}}{\sum_{i=1}^{N}\left(g_{i}+p_{i}\right)}$$

highlighting its utility in directly optimizing for segmentation accuracy.

Tversky Loss introduces an asymmetric factor to the traditional Dice framework, allowing for fine-tuned control over the model’s sensitivity to false positives and negatives. This is crucial for addressing class imbalances by adjusting the model’s focus toward underrepresented classes.


(3) $$L_{Tversky}=1-\frac{TP}{TP+\alpha FP+\beta FN}$$

Boundary Loss targets the precise delineation of object contours, a critical aspect in medical imaging, where accurate boundary identification can significantly impact diagnostic outcomes. This loss function seeks to minimize the distance between the predicted and actual boundaries, enhancing the model’s ability to capture detailed structural information.


(4) $$L_{Boundary}=\sum_{p\in P}\text{Φ}(p,By)+\sum q\in B_{y}\psi (q,P)$$

In summary, these loss functions each specialize in improving model performance for different aspects of tasks like segmentation, class imbalance, and boundary precision, especially in medical datasets (Equations 1–4).

### 2.4 Evaluation metrics

The performance of segmentation models is typically evaluated using a blend of volume based, distance-based metrics, and comparisons against expert variability, each offering unique insights into the model’s capabilities.

Volume-based metrics like the Dice Similarity Coefficient (DSC) and Jaccard Index measure the overlap between the model’s predictions and the ground truth, providing a direct indication of segmentation accuracy.


(5) $$\text{DSC}=\frac{2\cdot \left|A\cap B\right|}{\left|A\right|+\left|B\right|}$$

where *A* is the set of predicted segmentation points, and *B* is the set of ground truth segmentation points.

Distance-based metrics, including Mean Surface Distance (MSD) and Hausdorff Distance (HD), evaluate the geometric fidelity of the predicted segmentation, assessing the maximum discrepancy between the model’s output and the ground truth boundaries.


(6) $$\text{MSD}=\frac{1}{\left|S\right|+\left|T\right|}\left(\sum_{s\in S}d\left(s,T\right)+\sum_{t\in T}d\left(t,S\right)\right)$$

where *S* and *T* are the sets of surface points of the predicted and ground truth segmentations, respectively, and *d*(*x,Y)* denotes the shortest distance from point *x* to set *Y*.


(7) $$\text{HD}\left(S,T\right)=\text{max }\left\{\underset{s\in S}{\sup}\;d\left(s,T\right),\underset{t\in T}{\sup}\;d\left(t,S\right)\right\}$$

Expert variability comparisons gauge the model’s performance relative to human experts, using metrics such as inter- and intra-observer variability. This comparison helps contextualize the model’s accuracy within the bounds of human performance, offering a pragmatic assessment of its practical utility.

In synthesizing these elements, it becomes evident that the interplay between well-designed loss functions and robust evaluation metrics—such as DSC, MSD, and HD shown in Equations 5–7—is crucial for driving advancements in medical image segmentation and ensuring clinical applicability. This synergy not only guides the model optimization process but also ensures the relevance and applicability of the developed models to real-world medical diagnostics and treatment planning.

## 3 Site specific advances in malignant lymph nodes segmentation

**Figure 8:**
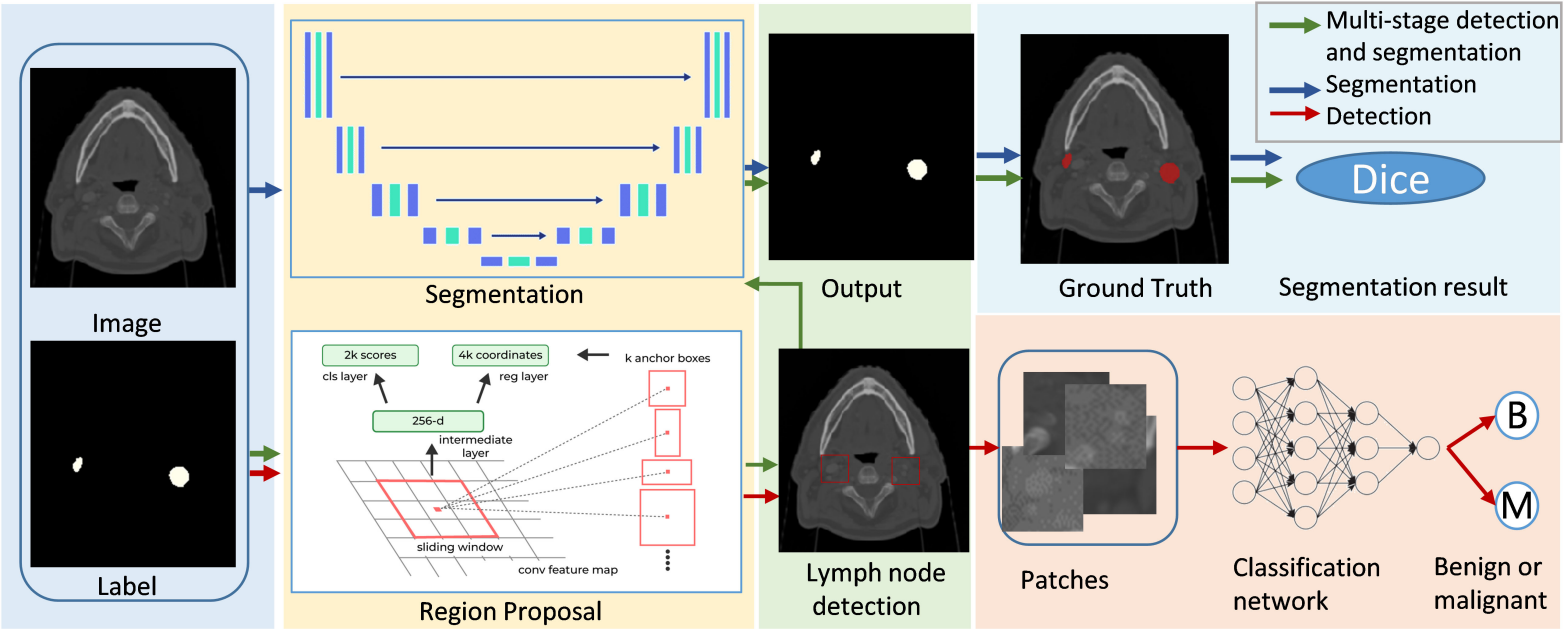
Deep learning lymph node detection and segmentation pipeline.

In the field of medical imaging, two fundamental tasks, detection and segmentation, play crucial roles but serve distinct purposes. Detection involves identifying the presence and location of objects, such as lymph nodes, within an image. Segmentation, on the other hand, goes a step further by delineating the exact contours of an object, providing more detailed spatial information about its shape and size. The main differences between segmentatoin and detection are listed in **Figure 7**. Malignant lymph node segmentation and detection have two primary pipelines: single-stage and multi-stage approaches, as shown in **Figure 8**.

In the single-stage pipeline, the focus is on directly identifying and segmenting malignant lymph nodes. This method classifies pixels into two categories: malignant lymph node pixels and other pixels. The primary advantage of this approach lies in its simplicity and directness, as it does not require intermediate steps or additional classification processes. This method is particularly effective when supported by high-quality annotated datasets, as it allows the model to learn the features of malignant lymph nodes more accurately.

The multi-stage pipeline, in contrast, involves a more complex procedure. Initially, this approach detects and segments all lymph nodes, distinguishing between lymph node pixels and non-lymph node pixels. Subsequently, the segmented lymph nodes are classified as benign or malignant using a neural network. This two-step process allows for a more refined analysis, as it isolates the task of lymph node detection from the classification of malignancy. The multi-stage pipeline can potentially achieve higher accuracy by focusing on different aspects of the problem in separate stages.

For segmentation tasks, the most commonly used network is the U-Net (27). It has gained widespread popularity due to its encoder-decoder structure, which efficiently captures context and spatial information. Notable benchmark methods include nnU-Net (51), which has set a high standard in medical image segmentation with its robust and adaptable architecture. Recently, the Segment Anything Model (SAM) (52) has gained attention for its versatility in various segmentation tasks. MedSAM (48), a specialized version of SAM tailored for medical applications, leverages SAM’s generalization capabilities and is fine-tuned on medical imaging data. This adaptation allows MedSAM to address the unique challenges of medical images, such as subtle anatomical variations and varying contrast levels, showing potential for becoming a significant tool in medical image segmentation.

Detection tasks often utilize sophisticated models like nnDetection (53) and Mask R-CNN (54). These models are designed to identify objects within images and have been adapted for the detection of lymph nodes. Additionally, some studies have employed U-Net (27) to generate candidates for detection, achieving remarkable results. By leveraging the strengths of segmentation networks to propose regions of interest, these methods can enhance the detection process, ensuring that candidate regions are thoroughly analyzed for the presence of malignant lymph nodes.

The segmentation and detection of malignant lymph nodes are essential components of modern medical imaging. However, defining a single best method is challenging, as model accuracy depends on the dataset. Various strategies can enhance generalizable lymph node identification and detection, including incorporating diverse, multi-institutional data to improve robustness, employing ensemble methods to combine different models for better performance, and leveraging self-supervised learning with advanced data augmentation techniques to enhance feature generalization. The following sections will discuss the segmentation and detection of malignant lymph nodes in various body regions, providing a detailed overview of recent work and advancements in each area.

### 3.1 Head and neck

The treatment of head and neck cancer (HNC) encompasses a range of modalities including surgery, radiation therapy, chemotherapy, and immunotherapy. Within this therapeutic framework, the automatic segmentation of tumors and metastatic lymph nodes plays a pivotal role. The effectiveness and prognosis of HNC treatment significantly depend on the local control of the tumor and the accurate identification and treatment of metastatic lymph nodes. However, the automatic segmentation of malignant lymph nodes in the head and neck region remains challenging due to the complex anatomy and the proximity of target volumes to organs at risk. **Table 3** summarizes related work on head and neck lymph node segmentation.

**Table 3:** Head and neck.

Publication	Year	Purpose	Image Modality	Patients	Ground Truth	Key Innovation	Outcome
Groendahl et al. (55)	2021	Tumors and involved nodes segmentation	PET/CT	197	Manual GTV delineation	Comparison of Traditional Methods with Machine Learning and Deep Learning Approaches	Dice = 0.75
Taku et al. (56)	2022	Involved LN segmentation	CT	110	Pathological Report	Use DL-CNN to segment GTVT for HPV-associated OPC	Dice = 0.92
Tekchandani et al. (57)	2022	LN segmentation and metastatic analysis	CT	175	Pathological Report	Introduced LNdtnNet: combines attention, residual UNet, squeeze-excitation for CT	Dice = 0.94
Wu et al. (58)	2022	Metastatic LN detection	CT	114	Pathologically Confirmed	Integrated lymph node features with GCN for top performance.	mFROC = 0.63
Bollen et al. (59)	2023	GTV segmentation	CT/PET/MRI	170	GTV delineation	Developed automated GTV delineation using 3D CNN with multimodal imaging	mDice = 0.89
Ariji et al. (60)	2022	Metastatic cervical LN segmentation	CT	158	Histopathological diagnosis	Utilize the U-Net architecture to segment metastatic cervical lymph nodes	F1 = 0.83
Liao et al. (61)	2025	Involved LN segmentation	CT	626	Manual segmentation	Used a two-stage transfer learning approach with nnUNet pretraining	Dice = 0.72
Al Hasan et al. (62)	2024	Small normal lymph nodes segmentation	CT	221	Manual segmentation	Enhanced Attentional U- Net by filtering encoder features for segmentation	Dice = 0.81

Medical imaging modalities such as Computed Tomography (CT), Magnetic Resonance Imaging (MRI), and Positron Emission Tomography/Computed Tomography (PET/CT) are commonly employed in the diagnostic and treatment processes of HNC. Numerous studies have focused on developing algorithms for the automatic segmentation of cancerous lymph nodes using these imaging modalities. As part of these efforts, Groendahl (55) and colleagues compared and evaluated conventional PET thresholding methods, six classical machine learning algorithms, and a 2D U-Net convolutional neural network (CNN) for automatic gross tumor volume (GTV) segmentation of HNC in PET/CT images. Their research was conducted on a dataset of 197 patients. The PET/CT-based CNN model demonstrated superior performance, with an average Dice coefficient of 0.75, compared to the best thresholding methods, which achieved an average Dice of 0.66, and classical machine learning models, which obtained an average Dice of 0.68. This validates the superiority of deep learning methods over traditional and machine learning approaches, with multi-modality models showing better results than those using a single modality.

The significance of 3D medical imaging is highlighted by Taku et al. (56), who demonstrated the successful auto-detection and segmentation of involved lymph nodes in HPV-associated oropharyngeal cancer using a 3-dimensional (3D) residual UNet architecture. Furthermore, Bollen (59) developed an automated GTV delineation approach for primary tumors and pathological lymph nodes based on a 3D CNN, exploiting multi-modality imaging(CT, PET and MRI) inputs as required in clinical practice. Their findings further validated the positive effects of multimodal fusion, especially PET, in enhancing segmentation performance.

Moreover, Tekchandani et al. (57) introduced a computer-aided diagnosis system for cervical lymph nodes in CT images, termed LNdtnNet. This system combines an architecture based on attention mechanisms and residual concepts with the base U-Net model (27) for detection, and employs a squeeze-andexcitation network for diagnosis. Wu et al. (58) integrated features from lymph node stations for metastatic lymph node detection by employing a GCN-based structure to model the relationships among different lymph node stations, achieving state-of-the-art performance in accuracy and sensitivity. Liao et al. (61) proposed a two-stage transfer learning approach for head and neck cancer (HNC) segmentation, leveraging a large-scale organ-at-risk (OAR) segmentation dataset to pretrain nnUNet, followed by fine tuning on a lymph node segmentation dataset. Their nnUNet-based method significantly improved segmentation performance, achieving a mean DSC of 0.72–0.74 and a mean HD95 of 2.73–3.78 mm, demonstrating enhanced model robustness and accuracy in HNC segmentation. Hasan et al. (62) proposed an Attentional U-Net-based approach for small lymph node segmentation, incorporating a feature filtering mechanism that enhances relevant contextual information from encoder features before integration with decoder features. Their method demonstrated high effectiveness in detecting and segmenting cervical lymph nodes measuring 5–10 mm, achieving a Dice score of 0.8084, highlighting its robustness in this challenging task.

In conclusion, the integration of advanced imaging modalities with innovative segmentation algorithms, particularly those leveraging deep learning techniques, offers significant potential for improving the accuracy and effectiveness of HNC treatment. The evolution of these technologies underscores the critical importance of precise tumor and lymph node segmentation in achieving favorable treatment outcomes for HNC patients.

### 3.2 Upper extremity

In the diagnosis and treatment of breast cancer, imaging modalities such as mammography, ultrasound, and MRI play critical roles. These technologies facilitate the identification and assessment of axillary lymph nodes (LNs), which are crucial for determining the stage and prognosis of the disease. Axillary LNs, including the apical axillary, interpectoral (Rotter’s), central axillary, lateral axillary, posterior axillary, and anterior axillary nodes, are key sites for the metastasis of breast cancer, occurring in approximately 33% of patients. These metastases significantly impact patient outcomes, making accurate lymph node segmentation a priority for effective treatment planning.

An overview of existing studies on upper extremity lymph node segmentation is provided in **Table 4**. Recent advancements in imaging analysis have introduced advanced algorithms to enhance the accuracy of LN segmentation and diagnosis. Zhang et al. (63) evaluated the Back-Propagation Neural Network (BPNN) algorithm for ultrasound image segmentation, demonstrating its superior diagnostic performance over conventional ultrasound methods in identifying axillary lymph node metastasis in breast cancer patients. The BPNN model exhibited greater specificity and a larger area under the curve (AUC) on the two-dimensional receiver operating characteristic (ROC) analysis compared to manual segmentation techniques.

**Table 4:** Upper extremity.

Publication	Year	Purpose	Image Modality	Patients	Ground Truth	Key Innovation	Outcome
Zhang et al. (63)	2021	LN segmentation and metastatic analysis	US	90	Pathological examination	Evaluated BPNN for ultrasound image segmentation	Acc = 0.97
Farfan Cabrera et al. (64)	2021	Pathological ALN segmentation	PET/CT	52	Manual delineation	Leveraging CT anatomy and PET function; enhancing CNN with C-Tree analysis	Dice = 0.87
Xu et al. (65)	2022	All ALN segmentation	US	216	Manual delineation	Novel bi-network with SAM and graph model enhances LN segmentation	Dice = 0.83

Additionally, Cabrera et al. (64) presented an innovative approach that combines CNNs with Component-Trees (C-Trees), building upon the U-Net architecture. This method utilizes a multi-modal U-Net, integrating PET and CT imaging data, along with a hierarchical model derived from PET scans to add high-level region based features as additional input channels. Their approach, validated against expert-defined ground truth, yielded promising outcomes, with segmentation achieving a Dice score of 0.867 and detection achieving a Dice score of 0.894.

To address challenges in ultrasound imaging, Xu et al. (65) introduced a novel bi-network architecture that incorporates a spatial attention module (SAM) and a graph based energy model with spatial attention constraints. This model is designed to enhance performance on complex images by providing additional discriminative information and capturing pixel relationships, significantly outperforming existing deep learning methods in LN segmentation tasks. Specifically, their approach improved segmentation accuracy by 5.14% compared to previous models.

These technological advancements underscore the importance of precise LN segmentation in breast cancer management, offering new avenues for improving the accuracy of diagnostics and the efficacy of treatment modalities such as radiotherapy and lymph node dissection (LND). As research progresses, these innovative imaging and segmentation techniques promise to refine our approach to breast cancer care, emphasizing the integration of advanced computational models for better patient outcomes.

### 3.3 Chest

In thoracic oncology, particularly lung and esophageal cancer, CT, PET, and MR imaging are crucial for diagnosis. Recent advances focus on thoracic lymph node segmentation to improve staging accuracy (see **Table 5** for a summary). Singh et al. (71) introduces a U-Net based method for generating candidate lymph nodes from chest CT volumes, employing different 3D representations to train CNNs, achieving 84% sensitivity with 2.88 false positives per volume. Mathai et al. (67) employ anatomical insights from 28 distinct structures to improve the performance, integrating these with lymph node labels to train three 3D nnUNet (51) models for automated segmentation. This approach attains a Dice score of 72.2 ± 22.3 for lymph nodes exceeding 8mm in size and 54.8 ± 23.8 for all lymph nodes, demonstrating the method’s efficacy in enhancing segmentation accuracy. Building upon these methods, Manjunatha et al. (68) propose a two-step approach for lymph node detection, starting with candidate generation via a modified U-Net with ResNet (72) to produce volumes of interest (VOIs), despite increasing false positives. This is followed by a reduction of these false positives using a 3D CNN classifier. The method achieves sensitivities of 87% at 2.75 FP/vol. and 79% at 1.74 FP/vol., illustrating its efficiency in detecting lymph nodes. Yan et al. (69) propose an end-to-end framework that enhances lymph node detection in esophageal cancer by leveraging station information with a multi-head detector, each focused on differentiating LN from non-LN structures at specific stations. Utilizing pseudo station labels for multi-task learning, this method notably increases detection sensitivity of thoracic lymph nodes to 71.4% and 85.5% at two false positives per patient, outperforming established methods like nnUNet (51), nnDetection, and LENS (73).

**Table 5:** Chest.

Publication	Year	Purpose	Image Modality	Patients	Ground Truth	Key Innovation	Outcome
Singh et al. (66)	2020	Enlarged LNs detection	CT	90	Manual delineation	U-Net based method for lymph node detection in chest CT volumes	Sen=0.84
Mathai et al. (67)	2024	Mediastinal LN segmentation	CT	104	Manual delineation	Utilize anatomical insights and lymph node labels for training 3D nnUNet models	Dice=0.72
Manjunatha et al. (68)	2023	Mediastinal LN detection	CT	90	Manual delineation	Two-step approach for lymph node detection: U-Net VOIs, 3D CNN	Sen=0.79
Yan et al. (69)	2023	LN detection	CT	173	Manual delineation	Propose end-to-end framework enhancing lymph node detection using multi-head detector	Sen=0.80&0.86
Bouget et al. (70)	2019	LN segmentation and malignancy analysis	CT	120	Manual delineation	2D pipeline combines U-Net segmentation, Mask R-CNN detection, tracks 3D	Dice=0.65

Bouget et al. (70) propose a 2D pipeline that combines U-Net’s pixelwise segmentation with Mask R-CNN’s instance detection, addressing data imbalance with a specific loss function and further refining pixel-wise labels through a final stage that leverages a tracking approach for 3D instance detection. This method, which represents detected instances with 3D masks, bounding volumes, and centroids, achieves an average Dice score of 76% across fifteen anatomical structures and a 75% recall at nine false positives per patient in lymph node detection, while maintaining an average centroid position error of 3mm in each dimension.

Guo et al. (74) introduce a segmentation framework that stratifies thoracic lymph node (LN) stations into three super stations and learns station-specific LN size variations. Conducting four-fold cross-validation on the NIH 89-patient dataset, this approach significantly surpasses prior works, yielding a 74.2% average Dice score, marking a 9.9% improvement, and a 72.0% detection recall, indicating a 15.6% enhancement, while reducing false positives to 4.0 per patient. Xu et al. (75) tackle LN segmentation challenges by introducing a cosine-sine (CS) loss function for voxel class imbalance and a multi-stage, multi-scale Atrous spatial pyramid pooling sub-module (MSASPP) into SegNet, termed DiSegNet (Dilated SegNet). These innovations lead to a marked improvement in performance, with DiSegNet achieving a 77% Dice similarity coefficient, surpassing the baseline SegNet’s 71% using cross-entropy loss.

### 3.4 Abdomen

The abdominal lymphatic drainage pathways run parallel to the blood vessels that supply or drain blood from organs, with many abdominal lymph nodes located in mesenteries, such as the mesentery of the intestine, mesocolon, and peritoneal ligaments, serving as potential sites for metastasis from gastric, liver, kidney, pancreatic, intestinal, or gallbladder tumors. However, the automatic segmentation of abdominal lymph nodes presents a significant challenge due to their high variability, low contrast, fuzzy boundaries, and clustering. To address this issue, researchers have proposed numerous innovative methods (see **Table 6**).

**Table 6:** Abdomen.

Publication	Year	Purpose	Image Modality	Patients	Ground Truth	Key Innovation	Outcome
Hoogi et al. (76)	2017	Pathological LN segmentation	CT	86	Pathologically confirmed	Fully-automated technique using machine learning for liver and lymph node lesion detection	Dice = 0.71
Zheng et al. (77)	2023	LN segmentation and metastasis prediction	CT	940	Pathologically confirmed	Enhances pancreatic LN segmentation using anatomical context and attention maps	AUC = 0.85
Bian et al. (78)	2023	LN segmentation and metastasis analysis	CT	734	Manual delineation	Introduced attention mechanism improves pancreatic LN localization and metastasis prediction	Acc = 0.85
Manjunatha et al. (68)	2023	Abdominal LN detection	CT	86	Manual delineation	Proposed two- stage lymph node detection with modified U-Net and 3D CNN	Sen = 0.80&0.86
Wang et al. (79)	2022	Abnormal LN Detection	MR	584	RECIST bookmarks on hand	Innovative MR detection network for LN identification using pseudo masks	AP25 = 0.55
Li et al. (80)	2021	LN segmentation	CT	176	Manual delineation	Presented a DRLLNS model using unsupervised RECIST-slices for segmentation	Dice = 0.77
Yu et al. (81)	2024	LN segmentation	CT	138	Manual delineation	Integrated LN-DDPM and nnUNet to enhance lymph node segmentation	Dice = 0.57

For instance Hoogi et al. (76) introduced a fully-automated technique leveraging machine learning and convolutional neural networks for the detection and segmentation of liver and lymph node lesions. When tested on CT scans featuring both liver lesions and pathological lymph nodes, this method demonstrated a detection sensitivity of 0.53 and a segmentation Dice score of 0.71 ± 0.15, underscoring its effectiveness and precision in such complex tasks. Furthermore, Zheng et al. (77) innovatively utilize anatomical spatial context and guiding attention maps from adjacent organs to enhance pancreatic lymph node (LN) segmentation, focusing segmentation on anatomically relevant areas and bypassing unlikely regions. Their approach integrates a pre-trained segmentation network with a new classification head for identifying metastasized LNs and employs a LN metastasis status prediction network that combines LN segmentation results with deep imaging features from tumors. Conducting extensive validation on a dataset of 749 pancreatic ductal adenocarcinoma (PDAC) patients and further external evaluations across two hospitals with 191 patients, their methodology significantly outperforms established benchmarks like nnUNet (51), CT-reported statuses, radiomics, and other deep learning models in LN detection and segmentation, achieving an improvement in accuracy by 1.8%.

To enhance malignant lymph node detection, Bian’s study (78) introduced an attention mechanism in a deep learning model, guided by the spatial context of surrounding organs and vessels, specifically targeting pancreatic LN locations. This approach, alongside a combined tumor and LN patient-level metastasis status prediction model based on automated LN segmentation and tumor annotations, outperformed conventional methods. After comparing with clinical and radiomics models, the deep learning approach emerged as the most effective, demonstrating superior accuracy in LN metastasis detection. Manjunatha et al. (68) proposed a two-stage approach for lymph node detection, beginning with candidate generation via a modified U-Net with ResNet architecture to identify volumes of interest (VOI) with high sensitivity, albeit increasing false positives. The subsequent stage employs a 3D CNN classifier for false positive reduction, complemented by an analysis of different deep learning models’ performance on decomposed 3D VOI representations. The methodology demonstrated efficacy on mediastinal and abdominal LN datasets, achieving sensitivities of 87% and 79% with manageable false positives per volume.

Building on this, Wang and colleagues (79) introduced an innovative detection network for MR image-based abnormal LN identification, utilizing pseudo masks from RECIST bookmarks for training instead of extensive pixel-wise annotations. Their network enhances the Mask R-CNN framework with global-local attention for context encoding and a multi-task uncertainty loss to balance multiple objectives, resulting in superior performance across a diverse abnormal abdominal LN dataset. Yu et al. (81) proposed a pipeline integrating a conditional diffusion model (LN-DDPM) for lymph node generation with nnUNet for segmentation, enhancing abdominal lymph node segmentation through realistic synthetic data. Their method, leveraging global and local conditioning mechanisms, achieved superior synthesis quality and improved segmentation performance, reaching a Dice score of 0.57 on abdominal lymph node datasets.

Further advancing LN detection techniques, Li (80) presented a deep reinforcement learning-based lymph node segmentation (DRLLNS) model, leveraging unsupervised segmentation of RECIST-slices for pseudo ground truth generation. This novel DRLLNS model, integrating U-Net with a policy network, optimizes LN bounding boxes and segmentation outcomes, showcasing remarkable performance against traditional image segmentation networks on a thoracoabdominal CT dataset in terms of Dice similarity coefficient and IoU metrics.

### 3.5 Pelvis

Pelvic lymph nodes (LNs), encompassing obturator, sacral, common iliac, external iliac, and internal iliac nodes, are critical for assessing the spread of pelvic urogenital or gastrointestinal tumors. LN staging plays a pivotal role in evaluating disease progression and guiding treatment decisions, as a high LN ratio (LNR) correlates with poorer survival outcomes. Furthermore, the International Federation of Gynecology and Obstetrics (FIGO) has developed a staging system for gynecologic malignancies, underscoring the importance of LN evaluation. Despite the critical role of LN metastasis status in patient management, the precise automatic segmentation of pelvic LNs remains challenging due to factors such as image intensity inhomogeneity, poor contrast, noise, and sensitivity to initial point selection. These issues highlight the need for advanced solutions in LN analysis to improve diagnostic and therapeutic strategies. A comparative overview of pelvic lymph node segmentation techniques is presented in **Table 7**.

**Table 7:** Pelvis.

Publication	Year	Purpose	Image Modality	Patients	Ground Truth	Key Innovation	Outcome
Zhao et al. (82)	2020	Pelvic LN detection and segmentation	MR	293	Manual delineation	Employed fused T2- weighted and diffusion- weighted images for LN analysis	Sen = 0.80
Bnouni et al. (83)	2018	LN Segmentation and Classification	MR	10	Manual delineation	Enhances PLN segmentation/classification using ensemble preprocessing and MRI fusion	Dice = 0.71
Liu et al. (84)	2021	Pelvic LN detection and segmentation	MR	393	Manual delineation	Investigates 3D U-Net efficacy in automating lymph node segmentation in DWI	Dice = 0.85
Wang et al. (85)	2021	Pelvic LN detection	CT	236	Manual delineation	Introduces two-level calibration and a novel keyframe- lymph node detection system	mFROC = 0.48
Wen et al. (86)	2024	Pelvic LN segmentation	CT	160	Manual delineation	Developed CMU-net for multi- head LNR classification and segmentation	Dice = 0.85

Zhao (82) employed fused T2-weighted and diffusion-weighted images to feed into Mask R-CNN via transfer learning, creating the auto-LNDS model for lymph node (LN) analysis. Validated on internal and external datasets of 935 and 1198 LNs respectively, the model outperformed junior radiologists in detection, with sensitivities of 80.0% and 62.6%, and PPVs of 73.5% and 64.5%, on the internal and external datasets, respectively. For LN segmentation, it achieved a Dice similarity coefficient (DSC) of 0.81-0.82, showcasing its efficacy and precision.

Bnouni et al. (83) present a computer-aided framework for enhancing pelvic lymph node (PLN) segmentation and classification by integrating ensemble preprocessing methods, iterative correction of initial segmentation points, and fusion of MRI modalities. They also utilize morphological and intensity features from segmented PLNs for accurate classification. This approach significantly improved segmentation accuracy from 61.37% to 66.53% (p¡0.05) and achieved a classification accuracy of 78.50% in distinguishing suspect from non-suspect PLNs, showcasing its effectiveness in PLN analysis.

Liu et al. (84) investigate the efficacy of the 3D U-Net algorithm in automating the detection and segmentation of lymph nodes (LNs) in pelvic diffusion-weighted imaging (DWI) for patients suspected of prostate cancer (PCa). Segmentation accuracy was evaluated against manual annotations of pelvic LNs. Initial results demonstrated a high Dice score of 0.85 for the segmentation of suspicious LNs, indicating promising performance of the 3D U-Net in LN detection and segmentation in DWI images of PCa patients. Similarly, Wang et al.’s (85) research introduced a two-level annotation calibration alongside a novel system that combines keyframe localization and lymph node detection networks, leveraging spatial and anchor priors for CT image analysis. Demonstrating significant improvements on PLNDataset and CTLNDataset, this approach outperforms existing methods and holds promise for clinical application in enhancing pelvic lymph node detection accuracy. Wen et al. (86) proposed a multi-head classification and segmentation framework, CMU-net, for pelvic lymph node region (LNR) detection and segmentation, leveraging ResNet-50 for classification and U-Net++ for segmentation. Their approach effectively addressed overlapping segmentation issues and improved spatial understanding, achieving DSC scores between 0.851 and 0.942, demonstrating high accuracy and clinical applicability.

These studies have utilized deep learning techniques, such as Mask R-CNN that integrates multi-modal images, methods that iteratively refine initial segmentation points, and 3D U-Net algorithms, achieving significant advancements in the automatic segmentation and detection of pelvic lymph nodes (LNs). These findings demonstrate that deep learning methods have significant potential in overcoming traditional challenges and improving the accuracy of automatic segmentation and detection of pelvic lymph nodes, having a positive impact on the integration into clinical workflows and patient prognosis.

### 3.6 Universal malignant lymph node detection

**Table 8** provides a summary of key methods developed for universal lymph node segmentation. Mathai et al. (87) propose an automated lymph node detection method in T2 MRI using neural network ensembles. By employing multiple state-of-the-art detection networks and bounding box fusion techniques, the method significantly reduces false positives and boosts detection accuracy. On a test set of 376 T2 MRI scans, the method achieved a precision of 71.75% and a sensitivity of 91.96% with 4 false positives per image. Subsequently, they (88) propose a universal computer-aided detection and segmentation pipeline that leverages T2 fat-suppressed (T2FS) and diffusion-weighted imaging (DWI) series from multiparametric MRI (mpMRI) studies. The T2FS and DWI series from 38 patients were co-registered and augmented, after which a Mask RCNN model was trained for universal 3D lymph node detection and segmentation. The experiments on 18 test mpMRI studies showed the proposed pipeline achieved a precision of 58%, sensitivity of 78%, and a dice score of 81%.

**Table 8:** Universal.

Publication	Year	Purpose	Image Modality	Patients	Ground Truth	Key Innovation	Outcome
Mathai et al. (87)	2023	LN detection and segmentation	MR	56	Radiology report	Employed a Mask RCNN model for universal 3D lymph node detection and segmentation	Dice = 0.81
Mathai et al. (87)	2023	LN detection	MR	376	Radiology report	Employed various deep learning detectors and improved detection performance through weighted box fusion	Dice = 0.81

Hou et al. (89) propose a deep learning-based automatic clinical target volume (CTV) segmentation method, clinically evaluated on multi-site tumor data from a single institution. The study involved 577 patients with nasopharyngeal, esophageal, breast, cervical, prostate, and rectal carcinomas, assessing Flexnet, Unet, Vnet, and Segresnet models. The results demonstrated high geometric consistency between auto-segmented and manually contoured CTVs, with most patients achieving Dice similarity coefficients (DSC) above 0.8. Additionally, 82.65% of auto-generated CTVs were either clinically accepted or required only minor revisions.

These studies highlight the progress in developing universal lymph node detection and segmentation methods using deep learning techniques. Despite these advancements, universal lymph node segmentation remains uncommon due to challenges such as anatomical variability across different body regions and the need for large, diverse datasets. Addressing these challenges presents a valuable direction for future research, as improved universal segmentation methods could significantly enhance diagnostic accuracy and treatment planning across various types of cancer.

## 4 Discussion

Despite advancements in deep learning, automatic segmentation of malignant lymph nodes remains fraught with significant challenges. Variability in imaging protocols poses significant challenges in medical imaging, particularly affecting the performance and generalizability of deep learning models (90). Differences in imaging parameters, device models, and scanning procedures across institutions lead to disparities in image quality and characteristics, which hamper a model’s ability to generalize effectively to new datasets. While standardizing imaging protocols is essential to mitigate these disparities, practical obstacles—such as the diversity of equipment manufacturers, variations in clinical workflows, and individualized patient needs—make this difficult to achieve. Additionally, accurately segmenting small targets with indistinct borders (58), like malignant lymph nodes, remains a substantial hurdle. The invasive nature of malignant cells blurs the boundaries between lymph nodes and surrounding tissues, complicating segmentation tasks even for advanced models (48). Moreover, the “black-box” nature of deep learning models raises concerns about interpretability and clinical applicability. Clinicians need to understand the rationale behind a model’s predictions to trust and effectively use them in patient care. Finally, models that perform well on training datasets may underperform when applied to data from different centers or imaging devices, limiting their widespread adoption. These challenges underscore the need for solutions that enhance model robustness, interpretability, and generalizability in varied clinical environments.

To address these challenges, future directions in the field are focusing on innovative approaches that can enhance the feasibility and effectiveness of deep learning models in clinical applications. Weakly supervised (91) and semi-supervised learning methods, which leverage unlabeled data to improve model performance, show promise in reducing the dependence on extensive annotated datasets. Enhancing model interpretability has also become a priority (92), as greater transparency can facilitate clinical decision-making and foster trust among healthcare professionals. Multimodal learning (93), integrating data from multiple imaging modalities such as CT, MRI, and PET, offers a more comprehensive view of malignant lymph nodes and has the potential to improve segmentation accuracy. Collaboration and standardization across healthcare centers are essential for enhancing model generalization; establishing common standards for data collection, preprocessing, and sharing can improve model robustness and facilitate application across diverse clinical scenarios. Additionally, federated learning (94) presents a viable solution for training models across multiple institutions without sharing raw data, addressing data privacy concerns and contributing to the advancement of deep learning techniques in medical image analysis. Collectively, these innovations hold promise for overcoming current limitations and advancing the field toward more reliable and clinically applicable models.

## 5 Conclusion

In conclusion, this paper has reviewed the advancements in deep learning-based segmentation and detection of malignant lymph nodes across multiple anatomical regions. Despite notable progress, challenges such as data limitations, model robustness, and variability in clinical conditions persist. Addressing these challenges will require not only further development of advanced algorithms but also the integration of AI into clinical workflows to achieve more automated, reliable, and precise analysis. The integration of innovative deep learning approaches holds the promise of not only enhancing the accuracy and efficiency of malignant lymph node detection and segmentation but also paving the way toward more personalized and effective cancer treatment strategies. By improving diagnostic accuracy and optimizing treatment planning, these technologies can help in reducing clinician workload and improving patient quality of care. As deep learning technologies evolve, they are expected to play a pivotal role in advancing oncological diagnostics and therapeutics, ultimately contributing to improved patient outcomes, more effective resource utilization, and a deeper understanding of cancer biology. Continued collaboration between AI researchers and clinicians will be essential to fully realize these potentials and bridge the gap between technical advancements and clinical needs.
